# Complement Inhibition in Coronavirus Disease (COVID)-19: A Neglected Therapeutic Option

**DOI:** 10.3389/fimmu.2020.01661

**Published:** 2020-07-07

**Authors:** Philip F. Stahel, Scott R. Barnum

**Affiliations:** ^1^Department of Specialty Medicine, College of Osteopathic Medicine, Rocky Vista University, Parker, CO, United States; ^2^CNine Biosolutions LLC, Birmingham, AL, United States

**Keywords:** COVID-19, coronavirus, hyperinflammation, innate immunity, complement inhibition, pharmacological treatment

## Introduction

The fatal outcome from coronavirus disease 2019 (COVID-19) is attributed to terminal respiratory failure secondary to bilateral pneumonia from severe acute respiratory syndrome coronavirus-2 (SARS-CoV-2) infection (1–3). However, a subset of younger patients with severe COVID-19 suffer from uncontrolled hyperinflammation and succumb to acute organ failure and cardiac arrest while still being adequately oxygenated (4–7). The “cytokine storm” or “cytokine release syndrome” (CRS) has been implicated in adverse patient outcomes, with interleukin-6 (IL-6) representing a key inflammatory mediator and surrogate marker of CRS (8, 9). The U.S. Food and Drug Administration (FDA) approved the expanded access to a recombinant monoclonal antibody against human IL-6 receptors (tocilizumab), and a randomized controlled phase 3 clinical trial on tocilizumab in adult patients suffering from severe COVID-19 is currently ongoing (10–13). The available empirical treatment modalities include a wide spectrum of off-label indications for antirheumatic agents, including cytokine inhibitors, corticosteroids, intravenous immunoglobulin, and other novel anti-inflammatory molecules (13–15). However, the exact mechanisms of hyperinflammation and hypercoagulation in COVID-19 patients remain enigmatic and poorly understood.

## Role of Complement in COVID-19?

The complement system serves as a “first line of defense” against invading viruses and as a bridge between innate and adaptive immune responses (16, 17). Interestingly, complement has received limited attention in the quest for effective anti-inflammatory treatment strategies in spite of multiple intuitive targets in COVID-19, and most of the prevalent anti-inflammatory agents currently under investigation do not include a consideration for complement inhibitors (8, 13, 14). Complement activation has been previously implicated in the pathophysiology of Middle East respiratory syndrome (MERS) and severe acute respiratory syndrome (SARS) which are severe infectious diseases mediated by coronaviruses that are similar to the pathogen responsible for the current COVID-19 pandemic (SARS-CoV-2). Experimental studies revealed that complement activation occurs in response to SARS-CoV infection, and mice deficient in genes for the central complement component C3 were shown to be protected from pulmonary inflammation and respiratory failure (18). Furthermore, the complement activation fragment anaphylatoxin C5a is a potent mediator of acute lung injury in highly pathogenic viral infections, including MERS and SARS (19). The pharmacological blockade of the C5a receptor (C5aR, CD88) attenuated pulmonary inflammation in a mouse model of MERS-CoV infection, and C5aR blockade led to decreased viral replication in infected lungs (20). In addition, there is an established crosstalk between the coagulation cascade and the immune proteolytic system through thrombin- and plasmin-mediated complement activation, and complement activation was recently postulated to induce thrombotic microangiopathy in COVID-19 (21, 22). In light of these presumed “key” pathophysiological features mediated by complement activation in response to coronavirus infections, it appears intuitive to consider the pharmacological complement inhibition as part of the “expanded access” paradigm to off-label indications for anti-inflammatory treatment strategies in COVID-19.

## Pharmacological Complement Inhibition

**Figure 1:**
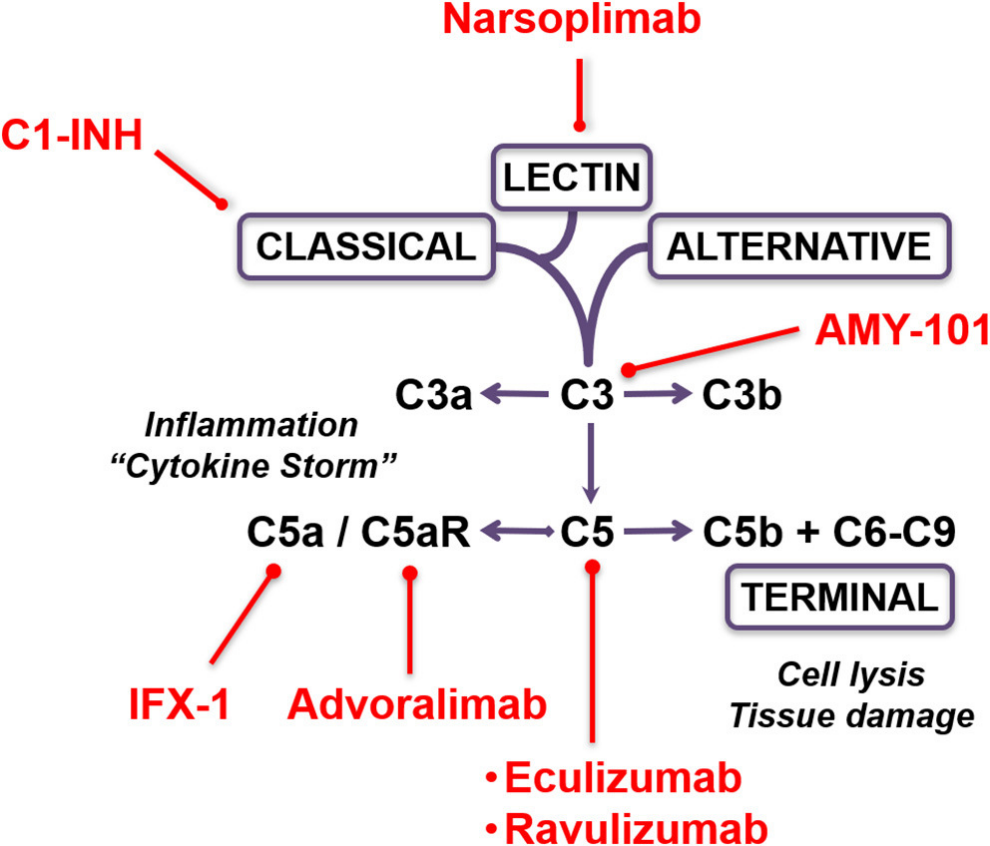
Therapeutic targeting options of the complement cascade in COVID-19. The schematic depicts the complement activation pathways and the sites of inhibition by the various pharmacological compounds.

There are currently multiple pharmacological complement inhibitors available for the treatment of rare inflammatory and autoimmune disorders in humans (17, 23, 24). Preliminary case reports from “hot zones” in Italy outlined the anecdotal success by compassionate use of the complement C3 inhibitor AMY-101 (Amyndas Pharmaceuticals, Glyfada, Greece) and by administration of the anti-C5 monoclonal antibody eculizumab (Soliris; Alexion, Boston, MA) in the rescue of critically ill COVID-19 patients (25, 26). From a mechanistic perspective, AMY-101 inhibits cleavage of C3, the central component in the complement cascade, and thus prevents the formation of the C3 and C5 convertases and the subsequent release of the inflammatory mediators C3a and C5a and formation of the tissue-damaging membrane attack complex (MAC; C5b-9). Further downstream, eculizumab prevents cleavage of C5 and the formation of the inflammatory anaphylatoxin C5a and of the MAC/C5b-9 (27). Indeed, a recent study from Milan, Italy, reported elevated levels of the C5 activation fragment C5a and soluble MAC (sC5b-9) in plasma samples of patients with severe COVID-19, confirming the notion that C5 blockade represents a potentially relevant therapeutic consideration (28). A prospective randomized controlled trial evaluating the safety and efficacy of eculizumab in patients with COVID-19 infection is currently under way (“CORIMUNO-19” trial). Several additional complement inhibitors are under consideration for compassionate use in COVID-19 (Figure 1). Of these, avdoralimab (Innate Pharma, Marseille, France) is an anti-C5aR monoclonal antibody that prevents binding of C5a to its receptor (C5aR, CD88), while IFX-1 (InflaRX; Martinsried, Germany) is a monoclonal antibody that targets C5a, preventing it from interacting with the C5aR. In addition, the recombinant human C1 esterase inhibitor conestat alfa (Ruconest; Pharming Group & Salix Pharmaceuticals, Bridgewater, NJ) is a specific inhibitor of the classical complement activation pathway which is currently approved for treatment of hereditary angioedema. This C1 inhibitor (C1-INH) is under consideration as an open-label, multicenter pilot trial in adult patients with SARS-CoV-2 pneumonia (“PROTECT-COVID-19” trial).

In addition to the classical and alternative complement activation pathways, the lectin pathway provides another new promising avenue for pharmacological targeting in COVID-19. The complement lectin pathway is activated when a pattern recognition receptor, such as mannose binding lectin (MBL), binds to pathogen-associated molecular patterns expressed on the surface of invading microorganisms (29). The complement cascade is then initiated by MBL forming complexes with MBL-associated serine proteases 1 and 2 (MASP-1 and MASP-2, respectively) in a similar fashion that C1 is activated through the classical pathway, leading to C4 and C2 cleavage and assembly of the C3 convertase (30). A historic case-control study on 569 SARS patients demonstrated a role of MBL gene polymorphisms in contributing to the susceptibility of viral invasion, and implied that the complement lectin pathway represents the “first line of defense” against SARS-CoV infection (31, 32). This notion is supported by recent histopathological findings in patients with severe COVID-19, demonstrating the deposition of complement lectin pathway components MBL and MASP-2, as well as complement activation fragments C4d and C5b-9, in the microvasculature of human lung tissue specimens with SARS-CoV-2 infection (21). Narsoplimab (OMS721; Omeros Corporation, Seattle, WA) is a novel human monoclonal antibody that targets MASP-2 and prevents lectin pathway-mediated inflammation and endothelial damage in a variety of autoimmune disorders (33). The safety and efficacy of narsoplimab is currently being investigated in a phase 2 dose-escalation cohort study in patients with complement-mediated hyperinflammatory conditions, including hematopoietic stem cell transplantation-associated thrombotic microangiopathy, thrombotic thrombocytopenic purpura, and atypical hemolytic uremic syndrome (33). Several other human complement inhibitors are currently in phase 2 or phase 3 clinical trials for different indications, raising the possibility that FDA-approved complement inhibitors will quickly join the developing arsenal of therapeutics for treatment of COVID-19 patients beyond compassionate use (34–36).

## Summary

In summary, the complex immune dysregulation observed in patients with severe COVID-19 remains poorly understood. The pharmacological targeting of complement activation in severe COVID-19 may attenuate the increased mortality observed in a younger cohort of patients with persistent hyperinflammation, thromboembolic complications, and cardiac arrest beyond terminal respiratory failure associated with SARS-CoV-2 pneumonia. Moving forward, it will be important to carefully monitor for beneficial and adverse effects associated with therapeutic complement inhibition (36). In addition, well-designed clinical studies are needed to determine patient outcomes by inhibiting complement in isolation vs. a combination therapy by targeting other key mediators responsible for the “cytokine storm” (37). Finally, from a patient safety perspective, we will have to determine the extent of retained innate immunity required for viral clearance and prevention of secondary bacterial infections.

## Author Contributions

Both authors conceived the concept of this article and wrote the manuscript together.

## Conflict of Interest

PS has a United States Patent No. 11,441,828 entitled: “Inhibition of the alternative complement pathway for treatment of traumatic brain injury, spinal cord injury, and related conditions.” PS is employed by HCA Healthcare as the chief medical officer at the Medical Center of Aurora. SB has a United States Patent # 10,535,004 “Methods and compositions for diagnosis and treatment of meningitis.” SB is employed by CNine Biosolutions LLC, a company involved in developing complement diagnostic assays. The authors declare that the research was conducted in the absence of any other commercial or financial relationships that could be construed as a potential conflict of interest. Specifically, there is no conflict of interest whatsoever by either of the two authors related to the pharmacological agents and companies cited in this manuscript.
